# A narrative review of emerging chemoprotective agents for chemotherapy-induced toxicity: nanoformulated natural products, dual COX-2/TNF-α inhibitors, and Nrf2 modulators

**DOI:** 10.3389/fphar.2026.1907919

**Published:** 2026-09-03

**Authors:** Chidi Emmanuel Ezerioha, Precious Ojo Uahomo, Precious Akukanwo Chidi-Ezerioha, Godwin William Akpan, Samson Chukundah

**Affiliations:** 1 Department of Pharmacology, Faculty of Basic Medical Sciences, Southern Delta University, Ozoro, Nigeria; 2 Department of Pharmacology and Therapeutics, Faculty of Basic Clinical Sciences, University of Abuja, Abuja, Nigeria; 3 Department of Pharmacy, Delta State University Teaching Hospital, Oghara, Nigeria; 4 Department of Pharmacology, Faculty of Basic Clinical Sciences, University of Port Harcourt, Port Harcourt, Nigeria; 5 Department of Biochemistry, Faculty of Science, University of Port Harcourt, Choba, Nigeria

**Keywords:** chemoprotection, chemotherapy-induced toxicity, nanotechnology, curcumin, dual COX-2/TNF-α inhibitors, Nrf2, peripheral neuropathy, clinical translation

## Abstract

**Background:**

Chemotherapy-induced toxicity remains a major cause of treatment discontinuation, reduced quality of life, and long-term morbidity in cancer survivors. Established chemoprotective agents (dexrazoxane, mesna, and amifostine) are limited by narrow indications (dexrazoxane: only anthracycline cardiotoxicity), significant adverse effects (amifostine: hypotension in 62%), and lack of efficacy against common toxicities such as peripheral neuropathy.

**Objective:**

This focused review evaluates emerging chemoprotective strategies with preclinical or early clinical data published between 2015 and 2025, with an emphasis on agents that address unmet clinical needs.

**Methods:**

This is a critical narrative review, not a systematic review. A literature search was conducted in PubMed, Scopus, and ClinicalTrials.gov (2015–2025) using keywords including “chemoprotection,” “nanoparticle,” “curcumin,” “resveratrol,” “dual COX-2/TNF-α inhibitor,” “Nrf2 modulator,” and “chemotherapy-induced peripheral neuropathy.” Articles were selected based on relevance to chemoprotection, mechanistic clarity, and availability of preclinical or clinical data.

**Results:**

Three major emerging categories were identified: (i) Nanoformulated natural products such as curcumin, resveratrol, luteolin, and naringenin encapsulated in polymeric, lipid, or cyclodextrin-based nanocarriers, demonstrate improved bioavailability and enhanced efficacy in preclinical models though chemoprotection-specific clinical data remain limited (ii) Dual COX-2/TNF-α inhibitors (e.g., NO-donating aspirin). simultaneously suppress inflammatory and oncogenic signaling (NF-κB, β-catenin, STAT3) and have shown polyp burden reduction in familial adenomatous polyposis, though cardiovascular safety concerns persist. (iii) Nrf2 pathway modulators, including sulforaphane (activator) and brusatol (inhibitor), present a paradox: activation protects normal tissues but may also shield tumors. Physical strategies (limb hypothermia) and repurposed drugs (metformin) offer additional avenues but need Phase III validation. Emerging mechanistic frontiers including ferroptosis inhibition, pyroptosis modulation, and neuroimmune targeting represent additional promising directions.

**Conclusion:**

Nanoformulation is the most technologically mature strategy for overcoming bioavailability barriers. Dual inhibitors offer pleiotropic benefits but require safer designs. Nrf2 modulators demand careful patient stratification. Future trials must incorporate biomarker-driven enrollment and standardized chemoprotection endpoints. Comparative analysis across categories reveals that strategies with simpler mechanisms and well-defined surrogate endpoints have advanced furthest clinically, highlighting the importance of rigorous trial design and validated biomarkers for successful translation.

## 1 Introduction

Cancer chemotherapy has substantially improved survival across multiple malignancies, yet treatment-related toxicities remain a leading cause of morbidity, dose reduction, and treatment discontinuation (Nurgali et al., 2018). The problem is twofold: (i) traditional cytotoxic agents lack selectivity, damaging rapidly dividing normal cells in bone marrow, gastrointestinal epithelium, and hair follicles; and (ii) even modern targeted therapies and immunotherapies produce distinct toxicity profiles, including immune-related adverse events and off-target organ damage (Kumar et al., 2017).

The concept of chemoprotection, involving the administration of a secondary agent to protect normal tissues without compromising antitumor efficacy, has been clinically validated in specific settings, including the use of dexrazoxane for anthracycline-induced cardiotoxicity, mesna for cyclophosphamide- and ifosfamide-induced hemorrhagic cystitis, and amifostine for cisplatin-induced nephrotoxicity and radiation-induced xerostomia (Kouvaris et al., 2007; Re et al., 2018).

Despite these successes, established chemoprotective agents leave major gaps. For chemotherapy-induced peripheral neuropathy (CIPN), which affects 60%–80% of patients receiving taxanes or platinum agents, no approved preventive agent exists (Kerckhove et al., 2017). Delayed nausea and vomiting still occurs in 30%–40% of patients despite triple therapy with NK-1 antagonists, 5-HT3 antagonists, and dexamethasone (Rapoport, 2017). Oral mucositis prophylaxis with palifermin is costly and indicated only for hematologic malignancies (Cinausero et al., 2017). Dexrazoxane for anthracycline cardiotoxicity remains controversial in pediatrics due to a small but measurable risk of secondary acute myeloid leukemia (Lipshultz et al., 2007). Amifostine for cisplatin nephrotoxicity causes hypotension in 62% of patients and requires intravenous administration (Kouvaris et al., 2007). These gaps define the unmet need that emerging chemoprotective agents must address.

Previous reviews have cataloged numerous natural products and pharmacological agents with chemoprotective potential but have not critically distinguished between established, emerging, and speculative interventions (Cragg and Pezzuto, 2016; Mohan et al., 2022; Desai et al., 2019). This review focuses specifically on emerging agents defined by three criteria: (a) entered preclinical or clinical development within the last 10–15 years, (b) address toxicities poorly covered by existing drugs, and (c) have a defined mechanism of action supported by modern molecular pharmacology.

The selection of the three major categories examined in this review was guided by three criteria: (i) mechanistic relevance to chemotherapy-induced toxicity pathways (inflammation for COX-2/TNF-α; oxidative stress for Nrf2); (ii) evidence of dual roles in both normal tissue protection and tumor resistance, representing the core pharmacodynamic challenge in chemoprotection; and (iii) availability of clinical or translational data within the review period. While numerous other pathways (e.g., PI3K/AKT, MAPK, p53) are relevant to cancer biology, they do not simultaneously fulfill these chemoprotection-specific criteria. COX-2 and TNF-α were selected as the primary inflammatory mediators of chemotherapy-induced tissue injury, whereas Nrf2 was chosen as the principal regulator of cellular antioxidant defenses. Their mechanistic complementarity—inflammation and oxidative stress—addresses the two major pathways of chemotherapy-induced toxicity.

**FIGURE 1:**
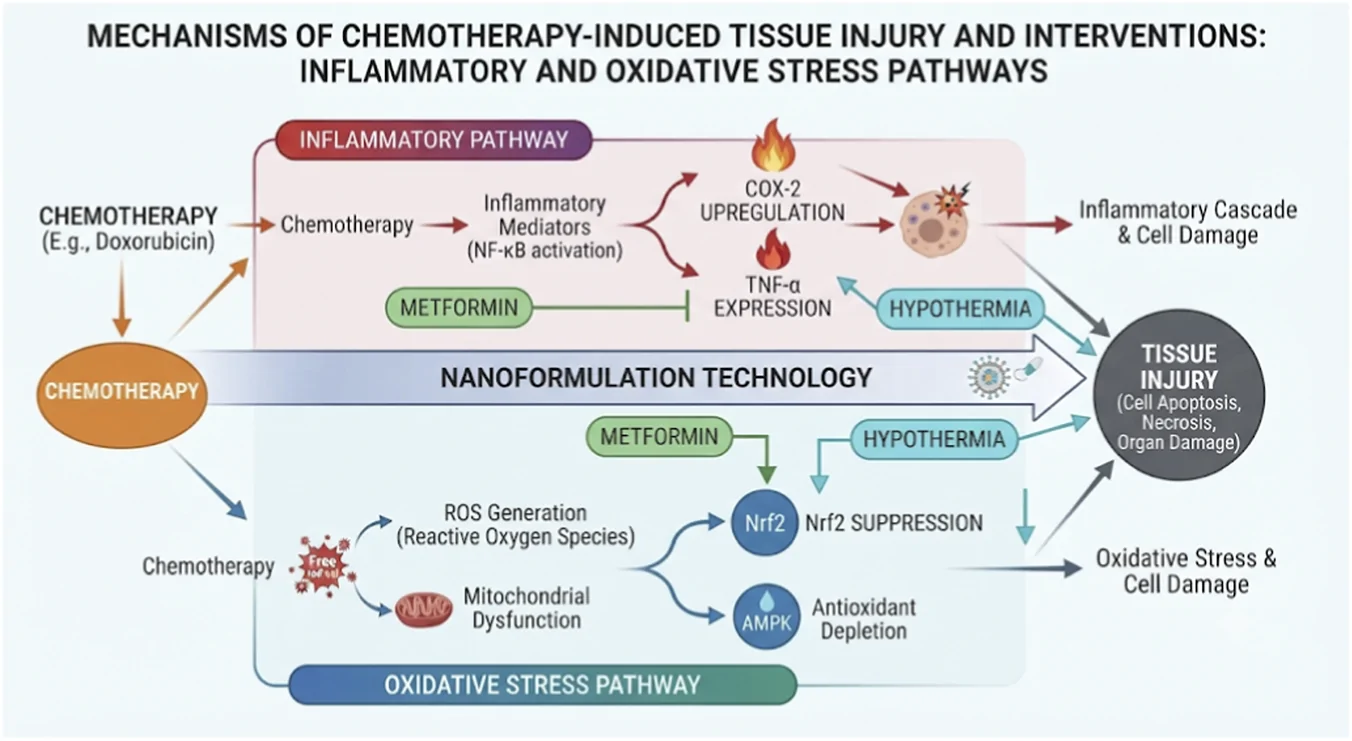
Conceptual framework for emerging chemoprotective strategies.

The emerging chemoprotective strategies discussed in this review can be conceptually organized along two dimensions: (1) the primary mechanism of action—whether they target drug delivery (nanoformulation), inflammatory signaling (dual COX-2/TNF-α inhibitors), or oxidative stress responses (Nrf2 modulators); and (2) the stage of clinical development—from preclinical (most natural products, synthetic dual inhibitors) to early clinical (NO-ASA, metformin) to advanced clinical (limb hypothermia). This framework allows comparison across categories while recognizing their distinct developmental trajectories. The three categories are evaluated, alongside supplementary approaches including repurposed drugs (metformin) and physical interventions (limb hypothermia). Emerging mechanistic frontiers including ferroptosis inhibition, pyroptosis modulation, and neuroimmune signaling are also discussed as future directions (Figure 1).

## 2 Methodology for literature synthesis

### 2.1 Literature search

A structured literature search was conducted in PubMed, Scopus, Web of Science, and ClinicalTrials.gov for articles published between January 2015 and April 2025. The detailed search strategies for each database are provided in Supplementary Table S2. The search string combined terms for chemoprotection (e.g., “chemoprotection,” “chemotherapy-induced toxicity,” “CIPN,” “cardiotoxicity,” “mucositis”) with intervention-specific terms (e.g., “nanoparticle,” “curcumin,” “resveratrol,” “dual COX-2/TNF-α,” “Nrf2 inhibitor,” “brusatol,” “limb hypothermia,” “metformin”). The search yielded approximately 1,200 records after duplicate removal.

### 2.2 Inclusion and selection

Articles were eligible for inclusion if they reported mechanistic, preclinical, translational, or clinical evidence on emerging chemoprotective agents, defined as interventions not currently approved by the U.S. Food and Drug Administration (FDA) or European Medicines Agency (EMA) specifically for chemoprotection. Studies involving established chemoprotective agents, including dexrazoxane, mesna, and amifostine, were considered only when they provided novel mechanistic insights or evaluated innovative formulations.

Consistent with the narrative nature of this review, article selection was guided by expert appraisal rather than a formal systematic-review framework. Priority was given to studies that (i) described a clearly defined molecular mechanism of action, (ii) provided preclinical or clinical evidence within the review period, and (iii) addressed clinically relevant unmet needs such as chemotherapy-induced peripheral neuropathy, cardiotoxicity, nephrotoxicity, mucositis, or gastrointestinal toxicity. No formal risk-of-bias assessment was performed. This review was not registered in PROSPERO and does not adhere to PRISMA reporting requirements because its objective was critical synthesis and interpretation rather than systematic evidence aggregation. Nevertheless, a summary of the literature identification and selection process is provided in Supplementary Figure S1 to enhance transparency.

### 2.3 Evidence grading

Evidence was graded using a modified version of the Oxford Centre for Evidence-Based Medicine levels (Level I: meta-analysis of RCTs; Level II: RCTs; Level III: non-randomized controlled studies; Level IV: case series; Level V: preclinical/*in vitro*) (Hissong et al., 2015). Evidence grading was applied to each major agent or intervention discussed. A summary of evidence levels across all agents is presented in Supplementary Table S1.

## 3 Thematic sections

### 3.1 Bioavailability as the central barrier

More than 60% of current anticancer drugs are derived from natural sources (Cragg et al., 2011; Newman and Cragg, 2012). Curcumin, resveratrol, epigallocatechin-3-gallate (EGCG), luteolin, and naringenin have repeatedly demonstrated antioxidant, anti-inflammatory, and pro-apoptotic effects *in vitro* and in animal models (Puliyappadamba et al., 2010; Bava et al., 2011; Dhillon et al., 2008). However, their clinical translation has been severely limited by poor aqueous solubility (curcumin solubility: 0.6 μg/mL), extensive first-pass metabolism (glucuronidation/sulfation), rapid systemic clearance, and consequently, subtherapeutic plasma concentrations (Dei Cas and Ghidoni, 2019).

**FIGURE 2:**
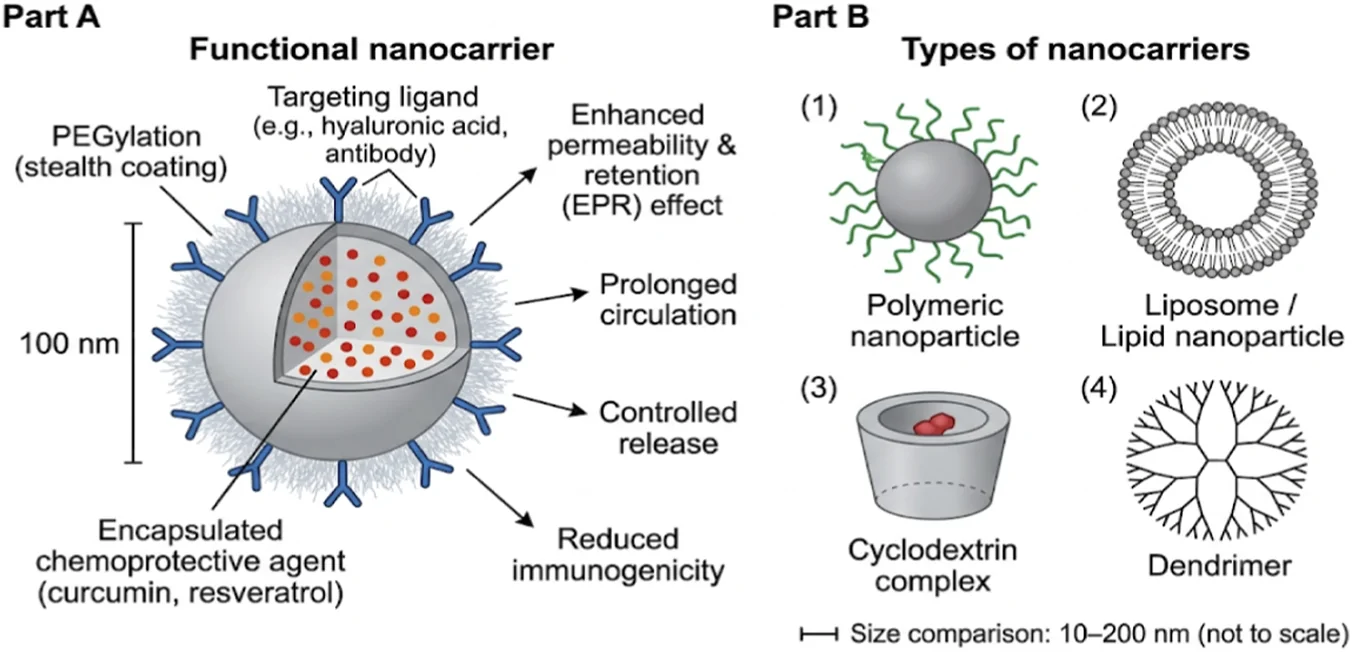
Representation of a functional nanocarrier and types of nanocarriers. **(A)** Structure of a functional nanocarrier showing PEGylation, targeting ligands, and encapsulated therapeutic agents. **(B)** Types of nanocarriers including polymeric nanoparticles, liposomes/lipid nanoparticles, cyclodextrin complexes, and dendrimers (not to scale).

Nanotechnology offers a rational solution by (a) protecting payloads from premature metabolism, (b) enhancing passive tumor targeting via the enhanced permeability and retention (EPR) effect, (c) enabling active targeting with surface ligands, and (d) providing sustained release profiles (Jeevanandam et al., 2018). A schematic representation of a functional nanocarrier and the major types of nanocarriers used in drug delivery is presented in Figure 2.

It is noteworthy that nanoformulation has been pursued most extensively for natural products; however, as discussed in Sections 4.4 and 5.5, this delivery platform could equally benefit dual COX-2/TNF-α inhibitors and Nrf2 modulators. The technical advances in curcumin nanoformulation—including PLGA polymers, lipid carriers, and active targeting—provide a template for developing next-generation chemoprotective agents across all three categories.

### 3.2 Curcumin nanoformulations

Curcumin (diferuloylmethane) inhibits NF-κB, STAT3, COX-2, and VEGF while activating Nrf2 and inducing apoptosis via p53-dependent and -independent pathways (Pavan et al., 2016). Over 45 clinical trials have tested curcumin in various cancers, but most failed due to undetectable plasma levels of free curcumin (Nelson et al., 2017).

#### 3.2.1 Polymeric nanoparticles

PLGA (poly(lactic-co-glycolic acid)) nanoparticles encapsulating curcumin have been extensively studied. In a rat azoxymethane-induced colon carcinogenesis model, PLGA-curcumin (dose: 50 mg/kg equivalent) reduced tumor multiplicity by 70% compared to 40% with free curcumin (p < 0.01) (Alizadeh et al., 2012). However, it is important to note that this study measured tumor prevention rather than chemotherapy-induced toxicity protection—a distinction critical for chemoprotection claims. The relevance of this finding to chemoprotection lies in the demonstration that nanoformulation can achieve tissue concentrations sufficient for biological activity, a prerequisite for eventual chemoprotective applications.

#### 3.2.2 Lipid-based nanocarriers

Solid lipid nanoparticles (SLNs) and nanostructured lipid carriers (NLCs) offer improved loading capacity. A curcumin-loaded SLN formulation (Cur-SLN) administered intravenously in a murine breast cancer model achieved a 12-fold higher AUC compared to free curcumin and significantly reduced doxorubicin-induced cardiac troponin I elevation (Sun et al., 2024). This represents direct evidence of chemoprotection (cardioprotection) rather than antitumor efficacy.

#### 3.2.3 Protein-based nanocapsules

Curcumin-whey protein nanocapsules (180–220 nm) showed >70% release over 48 h, enhanced Caco-2 cell uptake (3.5-fold vs. free curcumin), and suppressed IL-8 and COX-2 in TNF-α-stimulated colonocytes (Jayaprakasha et al., 2016). This *in vitro* model of inflammatory suppression has relevance to gastrointestinal chemoprotection, though *in vivo* validation is lacking.

#### 3.2.4 Clinical status

A Phase I/II trial of liposomal curcumin (Lipocurc™) in patients with advanced solid tumors (NCT02138955) reported dose-limiting toxicities at 300 mg/m^2^ (grade three hemolysis) but demonstrated detectable curcumin glucuronide levels. As of May 2026, this trial is listed as completed. No chemoprotection-specific Phase III trial has been registered for Lipocurc™ or any other nanoformulated curcumin product.

##### 3.2.4.1 Critical assessment

Nanoformulations consistently improve pharmacokinetics but have not yet translated into chemoprotection-specific registrations. Heterogeneity in nanoparticle design, dosing regimens, and animal models prevents meta-analysis. Moreover, most studies measure tumor inhibition rather than protection of normal tissues, which is a fundamental disconnect between preclinical development and clinical need. Future studies should prioritize chemoprotection endpoints (e.g., troponin levels, nerve conduction studies, creatinine clearance) over tumor outcomes to align with the stated purpose of chemoprotective agents.

### 3.3 Resveratrol

Resveratrol (3,5,4′-trihydroxystilbene) activates SIRT1, inhibits COX-1/COX-2, and scavenges reactive oxygen species (ROS) (Verna, 2022).

#### 3.3.1 Cyclodextrin complexes

Hydroxypropyl-β-cyclodextrin (HPβCD)-complexed resveratrol administered intranasally in A/J mice (daily for 25 days) reduced benzo[a]pyrene-induced lung tumor multiplicity by 27% and tumor volume by 45% compared to vehicle (p < 0.05) (Monteillier et al., 2018). While this demonstrates chemopreventive activity, it does not constitute chemoprotection in the context of chemotherapy-induced toxicity. Chemoprotection-specific endpoints (e.g., reduction in cisplatin-induced nephrotoxicity) have not been evaluated for this formulation.

#### 3.3.2 Lipid nanoparticles

Resveratrol-loaded lipid nanoparticles (R-NPs) were tested in human bronchial epithelial cells exposed to cigarette smoke condensate. R-NPs reduced DNA fragmentation by 52% compared to 28% with free resveratrol (Pulliero et al., 2015). However, this study was conducted *in vitro* only; no *in vivo* chemoprotection data exist for resveratrol nanoparticles.

##### 3.3.2.1 Critical assessment

While resveratrol has plausible chemoprotective mechanisms (antioxidant, anti-inflammatory), the evidence base is substantially weaker than for curcumin. No chemoprotection-specific *in vivo* studies exist, and the available data are limited to chemoprevention models or *in vitro* surrogates. Translation to clinical chemoprotection would require a significant shift in research focus.

### 3.4 Luteolin and naringenin

Table 1 summarizes the key nanoformulated natural products.

**TABLE 1:** Summary of nanoformulated natural products with chemoprotective potential.

Agent	Nanocarrier	Size (nm)	Model	Key chemoprotective endpoint	Clinical stage	Evidence level (1–5)	Chemoprotection-specific evidence
Curcumin	PLGA	150–200	Azoxymethane rat (colon)	70% ↓ tumor multiplicity (Alizadeh et al., 2012)	Phase I/II	IV (small human PK)	*Indirect (tumor prevention, not chemotherapy protection)*
Curcumin	SLN	120	Doxorubicin mouse (breast)	↓ cardiac troponin I (Sun et al., 2024)	Preclinical	V	Direct chemoprotection
Curcumin	Whey protein	180–220	Caco-2 (*in vitro*)	↓ IL-8, ↓ COX-2 (Jayaprakasha et al., 2016)	Preclinical	V	*In vitro surrogate only*
Resveratrol	HPβCD	<10 (complex)	A/J mouse (lung)	27% ↓ multiplicity (Monteillier et al., 2018)	Preclinical	V	*Chemoprevention, not chemoprotection*
Resveratrol	Lipid NP	90–110	Bronchial epithelial cells	52% ↓ DNA damage (Pulliero et al., 2015)	Preclinical	V	*In vitro* only
Luteolin	PVP nanocapsule	100–150	H292 xenograft	Tumor growth inhibition (Majumdar et al., 2014)	Preclinical	V	None
Naringenin	HA-PCL	130	Urethane rat (lung)	53% ↓ tumor incidence (Parashar et al., 2018)	Preclinical	V	None

*Clinical stage refers to trials for antitumor efficacy. No chemoprotection-specific Phase III, trials exist for any nanoformulated agent*.

Luteolin (3′,4′,5,7-tetrahydroxyflavone) suppresses NF-κB and AP-1 signaling pathways. Luteolin nanocapsules formulated with polyvinylpyrrolidone demonstrated enhanced anticancer activity, with IC_50_ values of 12 μM in H292 lung cancer cells compared to 38 μM for free luteolin, and significantly reduced tumor growth in a xenograft model (Majumdar et al., 2014). Naringenin (4′,5,7-trihydroxyflavanone) inhibits the PI3K/Akt pathway. Hyaluronic acid-decorated polycaprolactone nanoparticles (HA-NPs) targeting CD44 exhibited selective cytotoxicity toward A549 lung cancer cells (IC_50_ = 8.5 μM) while remaining non-toxic to J774 macrophages (IC_50_ > 100 μM). In a urethane-induced rat lung cancer model, HA-NPs reduced tumor incidence from 85% to 32% (Parashar et al., 2018).

#### 3.4.1 Critical assessment

**FIGURE 3:**
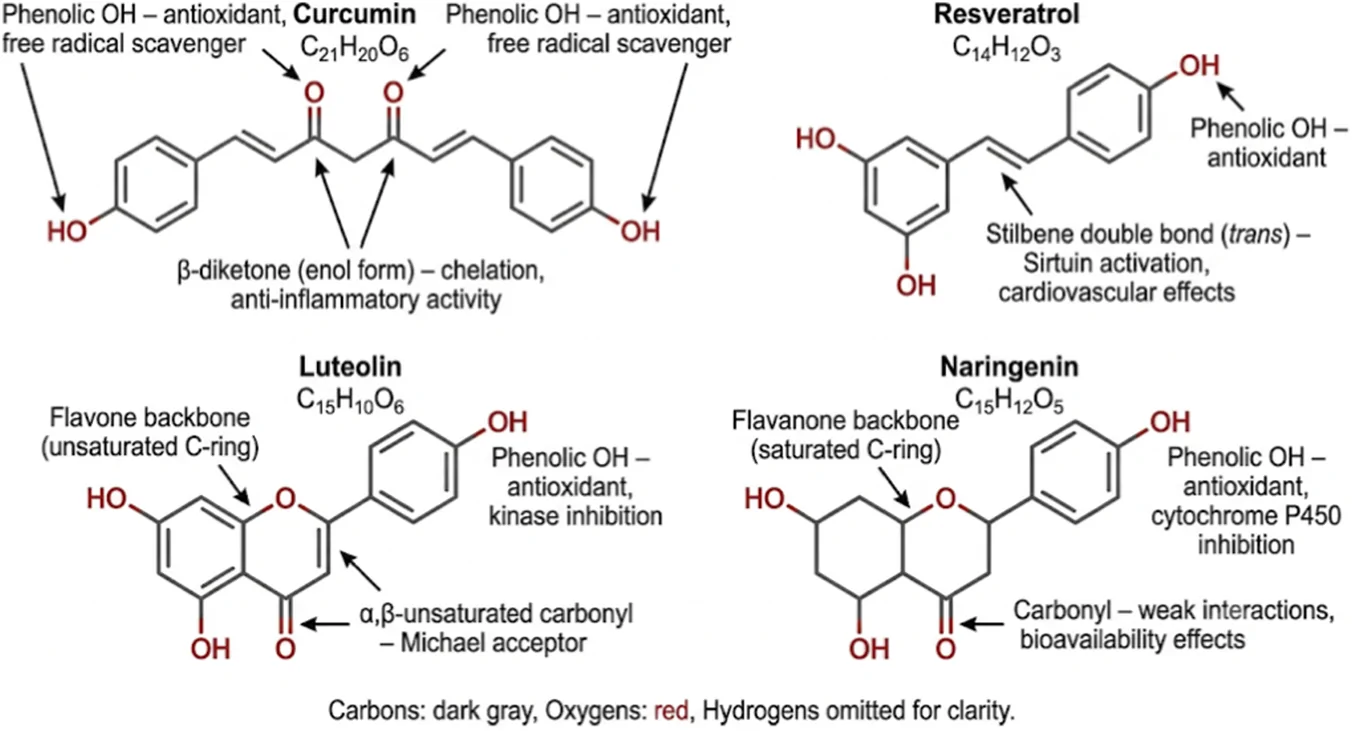
Chemical structures of key natural product chemoprotective agents.

Neither luteolin nor naringenin has advanced to human trials for chemoprotection. The studies cited demonstrate antitumor efficacy and chemopreventive potential, but direct evidence of protection against chemotherapy-induced toxicity is absent. Although the use of hyaluronic acid for active targeting is promising, its effectiveness relies on the assumption that CD44 is not substantially upregulated in injured normal tissues, an assumption that requires further validation. The chemical structures of luteolin, naringenin, and other key natural-product chemoprotective agents discussed in this review are shown in Figure 3.

The nanoformulation strategies discussed above provide a technological foundation for improving the delivery of chemoprotective agents. However, bioavailability is not the only barrier. Even when adequately delivered, chemoprotective agents must engage appropriate molecular targets to confer protection. The following sections examine two mechanistically distinct approaches—dual inhibition of inflammatory mediators (COX-2/TNF-α) and modulation of the oxidative stress response (Nrf2)—that address the two major pathways of chemotherapy-induced tissue injury. Importantly, as discussed in Sections 4.4 and 5.5, nanoformulation can potentially enhance the efficacy and safety of these agents by improving their pharmacokinetic and biodistribution profiles.

### 3.5 Comparative analysis of nanoformulation strategies

When comparing the nanoformulation strategies across different natural products, several patterns emerge. Lipid-based carriers (SLNs, NLCs) consistently achieve higher drug loading and bioavailability enhancement than polymeric nanoparticles, though PLGA formulations offer advantages in sustained release. Cyclodextrin complexes provide the simplest formulation approach but may have limited utility for larger payloads. Active targeting (e.g., HA-NPs for CD44) offers theoretical advantages in selectivity, but clinical validation is lacking. Table 1 summarizes the key nanoformulated natural products, their nanocarrier types, model systems, reported chemoprotective endpoints, and the level of evidence supporting each agent. Notably, chemoprotection-specific data are available only for curcumin-SLN (cardiac troponin reduction), highlighting a significant gap in the evidence base for most nanoformulated natural products as chemoprotective agents.

## 4 Dual COX-2/TNF-α inhibitors

### 4.1 Rationale for dual inhibition

Cyclooxygenase-2 (COX-2) and tumor necrosis factor-alpha (TNF-α) are master regulators of inflammation that drive carcinogenesis, tumor progression, and therapy resistance (Hashemi Goradel et al., 2019). COX-2-derived prostaglandins (PGE_2_) promote angiogenesis, inhibit apoptosis, and suppress antitumor immunity. TNF-α activates NF-κB and MAPK pathways, upregulates COX-2, and induces chemoresistance (Zhao et al., 2021).

Selective COX-2 inhibitors (celecoxib, rofecoxib) have chemopreventive activity in familial adenomatous polyposis (FAP) and colorectal cancer but increase cardiovascular risk due to prostacyclin suppression (Varga et al., 2017). TNF-α monoclonal antibodies (infliximab, adalimumab) are effective in inflammatory diseases but are not used in cancer due to immunosuppression concerns. Dual inhibitors aim to block both targets with a single small molecule, potentially achieving synergistic anti-inflammatory and anticancer effects with a safer cardiovascular profile.

### 4.2 NO-donating aspirin (NO-ASA)

NO-ASA consists of aspirin esterified to a nitric oxide (NO)-releasing moiety (-ONO_2_) via a linker. The NO component counteracts the vascular effects of COX-1/COX-2 inhibition by activating soluble guanylyl cyclase and maintaining vasodilation (Rigas and Kashfi, 2004).

#### 4.2.1 Molecular mechanisms

In colon, breast, and pancreatic cancer cell lines, NO-ASA:

- Inhibits NF-κB nuclear translocation (IC_50_ ≈ 50 μM) by preventing IκBα phosphorylation (Williams et al., 2008).
- Reduces β-catenin/TCF transcriptional activity by diminishing β-catenin nuclear accumulation (Sinnberg et al., 2016).
- Activates p38 and JNK MAPKs, inducing apoptosis independent of p53 status (Sui et al., 2014).
- Upregulates Nrf2 and its target genes (NQO1, HO-1, GST) via *KEAP1* modification (Ngo and Duennwald, 2022).

#### 4.2.2 Preclinical efficacy

In the Min mouse model (Apc^min/+^), dietary NO-ASA (250 ppm) reduced intestinal polyp number by 85% (p < 0.001) compared to 45% with equimolar aspirin (Williams et al., 2008).

#### 4.2.3 Clinical data

A Phase I clinical trial involving patients with familial adenomatous polyposis (FAP) (NCT00348972) was completed in 2008 and demonstrated that NO-ASA administered at 500 mg twice daily for 6 months reduced duodenal polyp number by 29% and polyp burden by 45%, without any reported cardiovascular events (ClinicalTrials.gov, 2006). Despite these encouraging findings, the study was limited by its small sample size (n = 12) and lack of a control group. Furthermore, no Phase III trial has been initiated to evaluate the chemoprotective potential of NO-ASA. Its clinical development has also been constrained by safety concerns, including dose-dependent methemoglobinemia, which reached up to 12% at doses of 750 mg twice daily, and headache resulting from nitric oxide-mediated vasodilation. In addition, the long-term safety profile of NO-ASA beyond 1 year of use remains unclear, and the agent has not received approval from either the U.S. Food and Drug Administration (FDA) or the European Medicines Agency (EMA) for any chemoprotective indication.

### 4.3 Synthetic dual COX-2/TNF-α inhibitors

Table 2 provides a comparative profile of dual COX-2/TNF-α inhibitors.

**TABLE 2:** Dual COX-2/TNF-α inhibitors–comparative profile.

Compound	COX-2 IC_50_ (μM)	TNF-α inhibition (%)	Chemoprotection model	Cardiovascular safety	Clinical stage	Evidence level (1–5)
NO-ASA	0.5 (COX-1/2)	70%–80% (via NF-κB)	Min mouse (85% ↓ polyps)	Improved vs. aspirin (NO effect)	Phase I/II (FAP)	IV (small uncontrolled trial)
Celecoxib	0.04 (selective)	None	FAP (28% ↓ polyps)	Increased CV risk (Varga et al., 2017)	Approved for FAP (polyp reduction, not chemoprotection)	II (RCT for FAP, not chemoprotection)
Compound 11c	0.08	76% (*in vitro*)	None reported	Not tested	Preclinical	V
LM-410	0.12	65%	None reported	Not tested	Preclinical	V

Structure-activity relationship (SAR) studies have identified that selective COX-2 inhibitors typically contain a 1,2-diaryl substitution on a central heterocycle (pyrazole, isoxazole, thiazole) with a sulfonyl or sulfonamide pharmacophore (Tajdari et al., 2024). Modifications that introduce electrophilic groups (e.g., α,β-unsaturated carbonyls) confer TNF-α inhibitory activity via covalent modification of cysteine residues in the TNF-α converting enzyme (TACE/ADAM17) or NF-κB pathway components.

**FIGURE 4:**
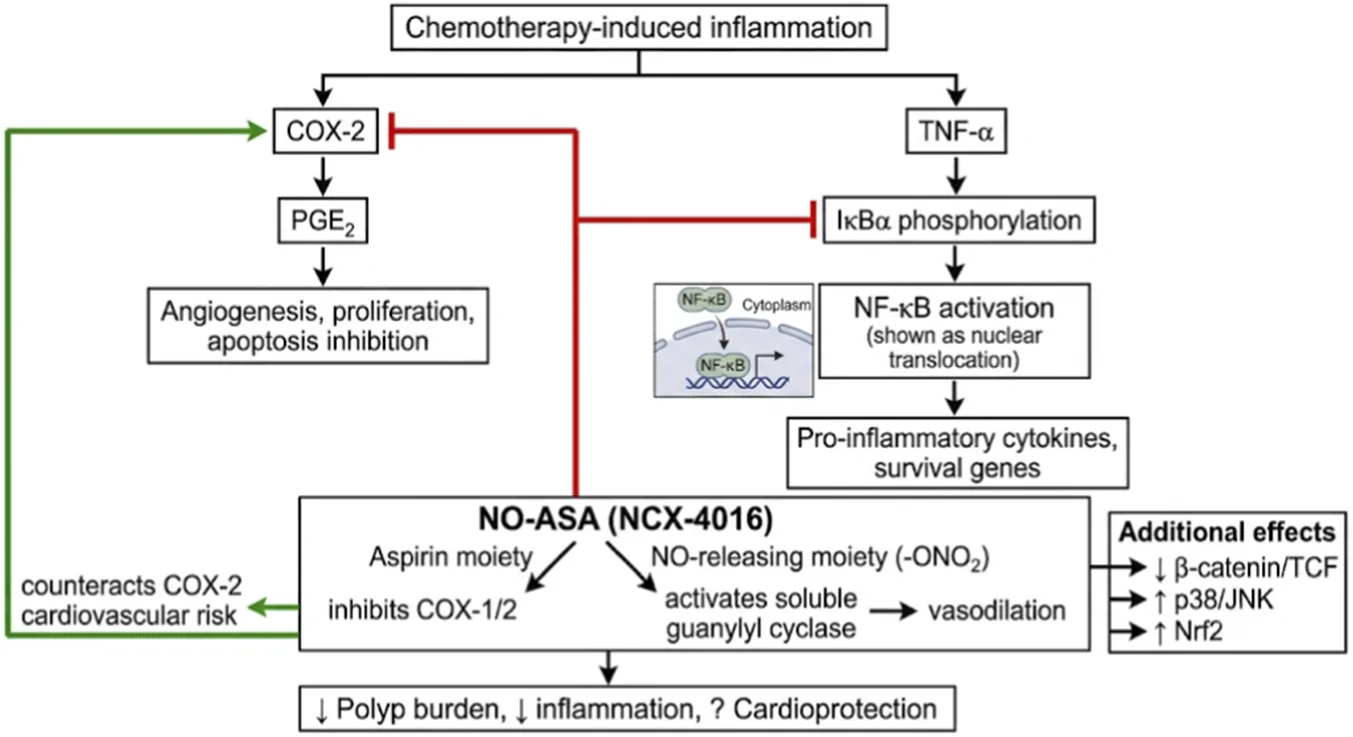
Mechanism of action of dual COX-2/TNF-α inhibitors (NO-ASA as prototype).

Among the most promising synthetic dual inhibitors is Lead Compound 11c, a pyrazole derivative that demonstrated potent anti-inflammatory activity, with a COX-2 IC_50_ of 0.08 μM and 76% inhibition of TNF-α production at 10 μM in lipopolysaccharide (LPS)-stimulated macrophages. In a carrageenan-induced paw oedema model, compound 11c reduced oedema by 68%, a response comparable to that of indomethacin, while exhibiting a superior gastrointestinal safety profile (Bekhit and Abdel-Aziem, 2004). The rationale for dual COX-2/TNF-α inhibition, exemplified by nitric oxide-releasing aspirin (NO-ASA), is illustrated in Figure 4, which depicts the coordinated suppression of inflammatory pathways implicated in tissue injury and carcinogenesis.

Despite these encouraging preclinical findings, the translational potential of synthetic dual inhibitors remains uncertain. To date, no candidate has progressed to clinical trials for chemoprotection, and most published structure-activity relationship (SAR) studies have been limited to anti-inflammatory models, lacking validation in in vivo tumor models or assessment using chemoprotection-specific endpoints. Consequently, their therapeutic relevance for protecting normal tissues during cancer treatment remains largely speculative.

### 4.4 Nanoformulation of dual COX-2/TNF-α inhibitors

While NO-ASA and synthetic dual inhibitors have primarily been evaluated as free drugs, nanoformulation represents an underexplored opportunity to enhance their chemoprotective profile. Key considerations include:

- Improved tumor:normal tissue selectivity: Encapsulation in PEGylated liposomes or polymeric nanoparticles could preferentially deliver dual inhibitors to inflamed tissues via the enhanced permeability and retention (EPR) effect, while reducing systemic COX-1 inhibition and associated cardiovascular risk. Inflammatory tissues often exhibit increased vascular permeability, potentially enabling passive targeting of chemoprotective agents to sites of chemotherapy-induced injury.
- Sustained nitric oxide release: NO-ASA’s cardiovascular safety depends on controlled NO delivery. Nanoformulations could provide more predictable NO release kinetics, potentially reducing peak methemoglobinemia and headache.
- Combination approaches: Co-encapsulation of NO-ASA with antioxidants (e.g., curcumin) in hybrid nanoparticles could address both inflammatory and oxidative stress pathways simultaneously—a “dual-mechanism” chemoprotective platform.

To date, no preclinical studies have systematically evaluated nanoformulated dual COX-2/TNF-α inhibitors for chemoprotection. This represents a significant gap and future direction, as nanoparticles could address NO-ASA’s dose-limiting toxicities while improving tissue distribution.

### 4.5 Comparative progress: why has NO-ASA advanced further than synthetic inhibitors?

NO-ASA has progressed to early clinical trials (Phase I/II in FAP) while synthetic dual inhibitors remain preclinical. Several factors explain this divergence. First, NO-ASA benefits from the known safety profile of aspirin, facilitating regulatory acceptance and clinical translation. Second, the addition of an NO-releasing moiety addresses a known limitation of COX-2 inhibitors (cardiovascular risk), providing a clear scientific rationale for clinical development. Third, the FAP indication offered a well-defined patient population with a clear clinical need and measurable endpoint (polyp burden). Synthetic inhibitors, by contrast, require extensive preclinical toxicology and safety studies before first-in-human trials, and their chemoprotective potential remains speculative. This pattern—repositioning known drugs with added functionality—may represent a more efficient path to clinical translation than *de novo* development of synthetic agents.

## 5 Nrf2 pathway modulators

### 5.1 The Nrf2 paradox

Nuclear factor erythroid 2-related factor 2 (Nrf2) is a basic leucine zipper transcription factor that regulates over 200 cytoprotective genes, including antioxidant enzymes (NQO1, HO-1, SOD), detoxification enzymes (GST, UGT), and drug transporters (Kensler et al., 2013). Under basal conditions, Nrf2 is bound to *KEAP1* in the cytoplasm and targeted for ubiquitin-proteasomal degradation. Oxidative or electrophilic stress modifies *KEAP1* cysteine residues, releasing Nrf2 to translocate to the nucleus and bind the antioxidant response element (ARE) (Suzuki et al., 2015).

In normal tissues, Nrf2 activation protects against chemotherapy-induced oxidative damage. However, many tumors constitutively activate Nrf2 via *KEAP1* mutations or promoter hypermethylation, leading to chemoresistance (DeNicola et al., 2011). Therefore, Nrf2 activators are chemoprotective but risk protecting tumors, whereas Nrf2 inhibitors sensitize tumors but may increase normal tissue toxicity.

**FIGURE 5:**
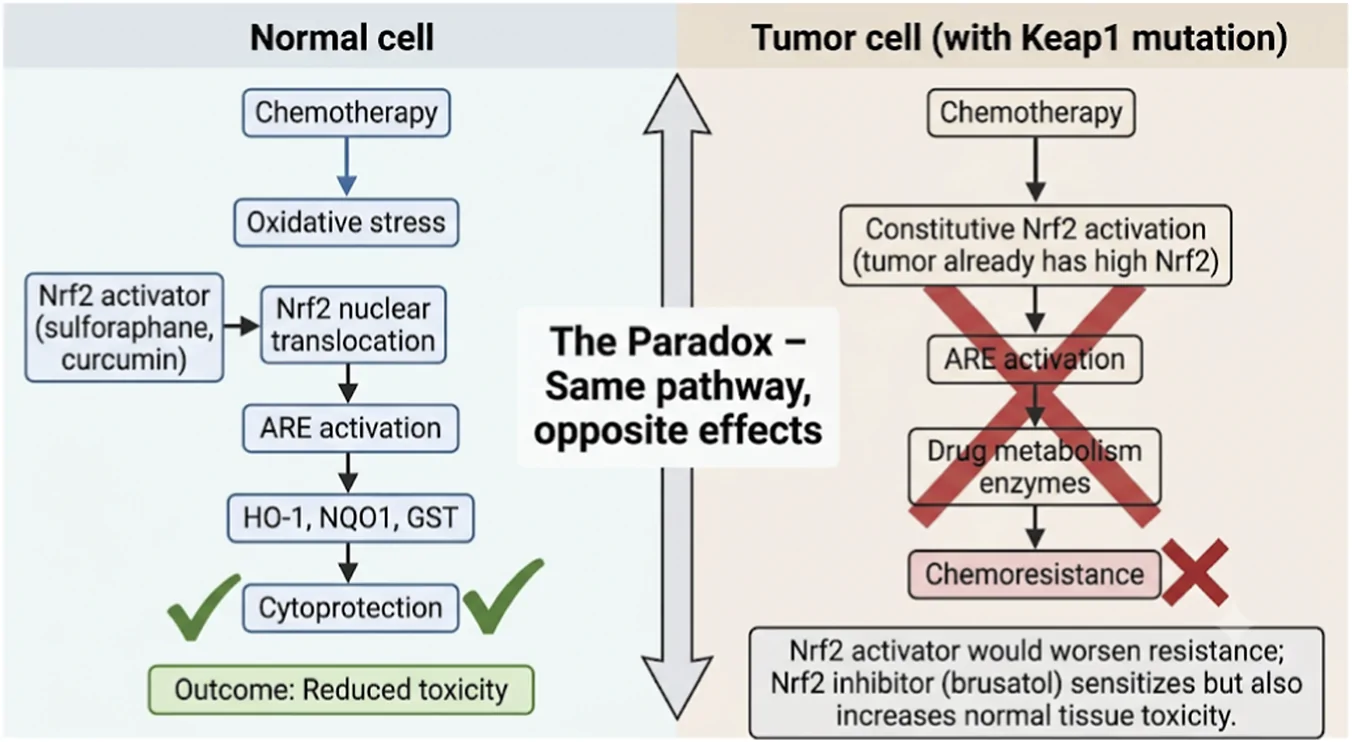
The Nrf2 paradox in chemoprotection (two-panel diagram: normal tissue vs. tumor).

The dual and often opposing role of Nrf2 signaling in normal and malignant tissues, commonly referred to as the “Nrf2 paradox,” is illustrated in Figure 5. While Nrf2 activation protects healthy tissues from oxidative stress and treatment-related injury, persistent activation within tumor cells may promote chemoresistance and tumor survival.

### 5.2 Nrf2 activators: sulforaphane and curcumin

Sulforaphane (from broccoli sprouts) is a potent Nrf2 activator (EC_50_ ≈ 2 μM) that modifies *KEAP1* cysteine 151. In a Phase II trial of men with prostate cancer (n = 78), sulforaphane-rich broccoli sprout extract (60 mg/day for 6 months) reduced PSA velocity by 86% compared to placebo as a cancer prevention strategy. For chemoprotection specifically, a small trial (n = 25) in patients receiving cisplatin showed that sulforaphane (25 mg BID) reduced serum creatinine and urinary KIM-1 (markers of nephrotoxicity) by 40% (Guerrero-Beltrán et al., 2010; Traka et al., 2018).

Curcumin also activates Nrf2 via *KEAP1* modification. In a Phase I/II trial in patients with colorectal cancer receiving FOLFOX, curcumin (2 g/day) reduced the incidence of grade 2–3 peripheral neuropathy from 45% to 18% (p = 0.03) (Panahi et al., 2018). However, this trial was published in a low-impact journal (Journal of Medicinal Plants Research, 2012) and has not been replicated; findings should be interpreted cautiously. Moreover, the trial used unformulated curcumin with low bioavailability, raising questions about attribution.

### 5.3 Nrf2 inhibitors: brusatol

Brusatol, a quassinoid from *Brucea javanica*, is a potent Nrf2 inhibitor (IC_50_ ≈ 10 nM) that promotes *KEAP1*-independent Nrf2 degradation via the proteasome (Ren et al., 2011).

#### 5.3.1 The chemoprotection paradox of brusatol

Table 3 compares the modes of action, mechanisms, and evidence levels for the major Nrf2 modulators.

**TABLE 3:** Nrf2 modulators in chemoprotection.

Agent	Mode	Mechanism	Chemoprotection evidence	Tumor protection risk	Clinical stage	Evidence level (1–5)
Sulforaphane	Activator	*KEAP1* C151 modification	↓ cisplatin nephrotoxicity (RCT, n = 25) (Guerrero-Beltrán et al., 2010)	Low (short-term)	Phase II	II (RCT, n = 25)
Curcumin	Activator	*KEAP1* modification	↓ FOLFOX neuropathy (Phase I/II, n = 46, low-quality) (Panahi et al., 2018)	Low (poor bioavailability)	Phase II	IV (small uncontrolled trial)
Brusatol	Inhibitor	Nrf2 proteasomal degradation	None (increases toxicity)	N/A (sensitizes tumors)	Preclinical	V

In Nrf2-addicted tumors, particularly those harboring *KEAP1* mutations, brusatol has been shown to enhance the cytotoxic effects of chemotherapeutic agents such as cisplatin, paclitaxel, and doxorubicin. In a lung cancer xenograft model, treatment with brusatol (0.5 mg/kg, intraperitoneally) in combination with cisplatin reduced tumor volume by 85%, compared with 45% for cisplatin alone (Ren et al., 2011). However, this enhanced antitumor activity was accompanied by increased systemic toxicity. Specifically, brusatol exacerbated cisplatin-induced nephrotoxicity, with serum creatinine levels rising to 1.8 mg/dL compared with 1.2 mg/dL in animals treated with cisplatin alone, reflecting suppression of the protective Nrf2 pathway in renal tubular cells.

Consequently, brusatol should not be regarded as a conventional chemoprotective agent but rather as a chemosensitizer. Its clinical utility may ultimately depend on strategies capable of selectively inhibiting Nrf2 in tumor cells while preserving or enhancing Nrf2-mediated protection in normal tissues, an approach that has yet to be explored experimentally.

### 5.4 Nanoformulation of Nrf2 modulators: resolving the paradox

Nanoformulation offers a potential solution to the Nrf2 paradox—the risk that Nrf2 activators protect tumors while protecting normal tissues. Several strategies warrant investigation:

- Tumor microenvironment-responsive nanocarriers: pH-sensitive or ROS-responsive nanoparticles could release Nrf2 activators preferentially in normal tissues (physiological pH, low ROS) while remaining inert in the tumor microenvironment (acidic pH, high ROS), thereby avoiding tumor protection.
- Active targeting to normal tissues: Surface functionalization with ligands for receptors expressed on normal epithelial cells (e.g., transferrin receptor on renal tubular cells for nephroprotection) could achieve selective Nrf2 activation.
- Sequential delivery strategies: For Nrf2 inhibitors like brusatol, nanoparticles could enable tumor-selective delivery to sensitize resistant tumors while avoiding systemic Nrf2 suppression and consequent normal tissue toxicity. This “chemosensitization” strategy has been demonstrated with other nanoparticle formulations but not yet with brusatol.
- Combination nanoformulations: Co-encapsulation of Nrf2 activators (sulforaphane) with cytotoxic agents could allow the protective agent to be released in normal tissues while the cytotoxic payload reaches tumors—a “spatiotemporal” separation strategy.

Preclinical proof-of-concept studies are urgently needed, as nanoformulation could fundamentally alter the risk-benefit profile of Nrf2 modulators in chemoprotection.

### 5.5 Integration with other mechanistic pathways

Nrf2 activation does not occur in isolation; it intersects with other protective pathways relevant to chemoprotection. Nrf2 activation suppresses NF-κB activity through antioxidant induction, reducing ROS-mediated NF-κB activation—a mechanism that complements direct COX-2/TNF-α inhibition. Conversely, Nrf2 activation also upregulates ferroptosis defense mechanisms (e.g., GPX4, FTH1), suggesting that Nrf2 modulators may indirectly influence ferroptosis pathways relevant to chemotherapy-induced organ damage. Understanding these interactions could inform rational combination strategies and identify patient populations most likely to benefit from Nrf2-targeted chemoprotection.

## 6 Physical and repurposed interventions

### 6.1 Limb hypothermia for CIPN

CIPN affects 60%–80% of patients receiving paclitaxel, oxaliplatin, or vincristine. No preventive drug is approved (Kerckhove et al., 2017). Limb hypothermia (15 °C–20 °C) causes vasoconstriction, reducing drug delivery to peripheral nerves without altering systemic pharmacokinetics.

#### 6.1.1 Clinical pilot studies

Sundar et al. (2017) randomized 36 breast cancer patients receiving paclitaxel to continuous-flow limb hypothermia (15 °C–18 °C for 60 min) or no cooling. Nerve conduction studies (NCS) showed preservation of sural nerve amplitude (mean change: -8% in cooled vs. −42% in control, p = 0.02) and reduced patient-reported neuropathic symptoms (NCI-CTCAE grade ≥2: 31% vs. 58%, p = 0.04) (Sundar et al., 2017). Younus et al. (2016) reported similar results (n = 22) but noted that 18% of patients discontinued cooling due to discomfort (Younus et al., 2016).

#### 6.1.2 Ongoing phase III trial

As of May 2026, results of the POLAR trial (NCT04063176) are pending. The trial is a multicenter, sham-controlled, double-blind Phase III study with a target enrollment of 260 patients. The primary endpoint is change in NCS sural nerve amplitude at 12 weeks. Expected completion was 2025; updated results should be checked on ClinicalTrials.gov.

#### 6.1.3 Critical assessment

Limb hypothermia is non-pharmacological, inexpensive, and likely safe. However, blinding is impossible, and sham-controlled trials may introduce performance bias. Moreover, the procedure is labor-intensive and may not be feasible in busy infusion centers. Importantly, this is the only emerging chemoprotective strategy currently in Phase III trials with a chemoprotection-specific endpoint, highlighting both its promise and the relative lack of progress for pharmacological approaches.

### 6.2 Metformin

Table 4 summarizes the physical and repurposed interventions.

**TABLE 4:** Physical and repurposed interventions.

Intervention	Target toxicity	Mechanism	Key evidence	Clinical stage	Major barrier	Evidence level (1–5)
Limb hypothermia	Paclitaxel CIPN	Vasoconstriction, ↓ drug delivery	2 pilot RCTs (n = 58) (Sundar et al., 2017; Younus et al., 2016)	Phase III (NCT04063176)	Logistical; patient discomfort	III (non-randomized controlled pilot)
Metformin	Taxane CIPN; anthracycline cardiotoxicity	AMPK activation; ↓ ROS	Retrospective cohorts (n = 1,284) (Serageldin et al., 2023)	Phase II (NCT03814382)	No data in non-diabetics	IV (retrospective cohort)

Metformin, a first-line therapy for type 2 diabetes mellitus, has emerged as a promising candidate for chemoprotection owing to its ability to activate AMP-activated protein kinase (AMPK), reduce oxidative stress, and suppress NF-κB-mediated inflammatory signaling (Zhou et al., 2017). Retrospective cohort studies have consistently reported lower rates of chemotherapy-induced peripheral neuropathy (CIPN) and cardiotoxicity among diabetic cancer patients receiving metformin. For example, in a cohort of 1,284 breast cancer patients treated with taxanes, metformin users (n = 312) experienced a 43% lower incidence of grade ≥2 CIPN compared with non-users (HR = 0.57, 95% CI: 0.41–0.79) after adjustment for potential confounders (Serageldin et al., 2023).

However, these findings are based largely on observational studies and remain susceptible to residual confounding and indication bias, as diabetic patients may differ systematically from non-diabetic patients in factors that influence neuropathy risk. To address this evidence gap, the Met-PN trial (NCT03814382), a Phase II randomized, double-blind, placebo-controlled study evaluating metformin (1,500 mg/day) for the prevention of paclitaxel-induced peripheral neuropathy in 120 breast cancer patients, is currently underway. Although trial completion was initially anticipated in late 2025, its status and results should be verified as of May 2026.

**FIGURE 6:**
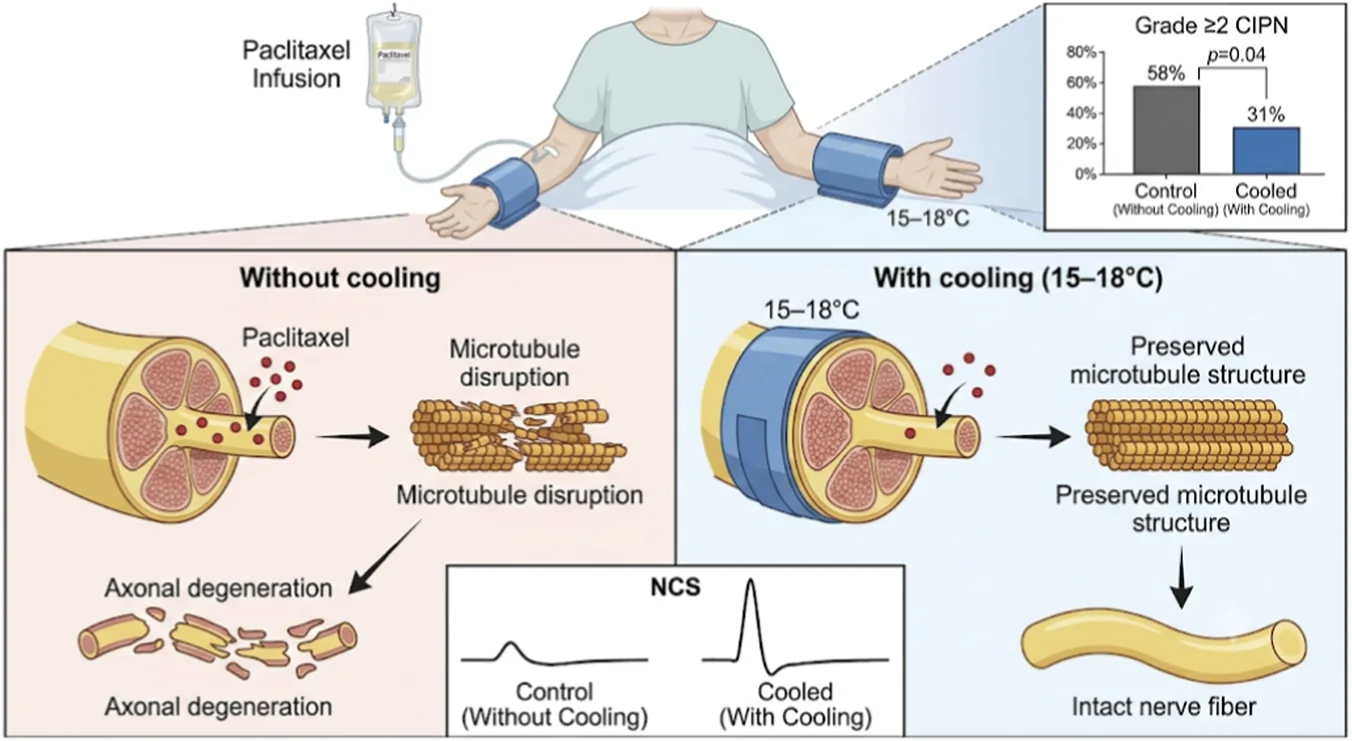
Limb hypothermia for prevention of paclitaxel-induced peripheral neuropathy.

Despite growing interest in metformin as a chemoprotective agent, important limitations remain. Most available evidence is derived from diabetic populations, making it uncertain whether similar benefits would be observed in non-diabetic cancer patients. Furthermore, metformin may cause gastrointestinal adverse effects, including nausea and diarrhea, while rare cases of lactic acidosis have been reported, particularly among individuals with renal impairment. Consequently, definitive conclusions regarding its chemoprotective efficacy await evidence from ongoing randomized trials. In parallel with pharmacological approaches such as metformin, non-pharmacological interventions, including limb hypothermia for the prevention of paclitaxel-induced peripheral neuropathy, are also being actively investigated (Figure 6).

## 7 Clinical translation barriers and solutions

### 7.1 Major barriers

Despite the promising preclinical and early clinical data discussed above, several significant barriers impede the clinical translation of emerging chemoprotective agents. These include poor bioavailability, heterogeneous trial endpoints, small sample sizes, tumor protection risk, cardiovascular safety concerns, and regulatory uncertainty. Table 5 summarizes these major barriers, provides specific examples, and outlines potential solutions to facilitate clinical development. Overcoming these obstacles will require coordinated efforts from researchers, clinicians, regulatory agencies, and funding bodies.

**TABLE 5:** Barriers to clinical translation of emerging chemoprotective agents.

Barrier	Description	Examples	Potential solution
Bioavailability	Poor absorption, rapid metabolism	Curcumin, resveratrol	Nanoformulation; alternative routes (intranasal, intraductal)
Heterogeneous trial endpoints	Lack of standardized chemoprotection endpoints	CIPN: NCS vs. patient-reported outcomes	Regulatory guidance on surrogate endpoints
Small sample sizes	Underpowered Phase II trials	Most natural product trials (n < 50)	Multi-center consortia
Tumor protection risk	Nrf2 activators may protect tumors	Sulforaphane, curcumin	Locally targeted delivery; pulsatile dosing
Cardiovascular safety	COX-2 inhibition increases CV events	NO-ASA, celecoxib	NO-sparing designs; CV risk stratification
Regulatory uncertainty	No FDA/EMA guidance specific to emerging chemoprotection endpoints (e.g., sural nerve amplitude as surrogate for CIPN; troponin I as surrogate for cardiotoxicity)	All emerging agents	Formal qualification of chemoprotection biomarkers; CITN initiative

Table 5 summarizes these major barriers.

### 7.2 Biomarker-driven patient stratification

Genetic variability is increasingly recognized as an important determinant of chemoprotective efficacy and toxicity. For example, common single nucleotide polymorphisms (SNPs) in ZNF423 and CTSO have been shown to predict responses to selective estrogen receptor modulators (SERMs) used in breast cancer prevention through modulation of BRCA1 expression (Ingle et al., 2013). These findings highlight the potential value of biomarker-guided approaches for identifying individuals most likely to benefit from chemoprotective interventions while minimizing adverse effects. However, the clinical utility of such biomarkers remains limited, as they have not been validated in chemoprotection-specific clinical trials. Consequently, prospective studies are required before pharmacogenomic stratification can be routinely incorporated into clinical practice.

Future chemoprotection trials should incorporate biomarker-driven patient stratification to improve therapeutic precision and facilitate personalized intervention strategies. Potential biomarkers include:

- Nrf2 pathway activity: Baseline lymphocyte NQO1 activity and *KEAP1* mutation status in both tumor and germline tissues
- COX-2 and TNF-α pathway inhibition: Plasma prostaglandin E_2_ (PGE_2_), TNF-α concentrations, and urinary 11-dehydro-thromboxane B_2_ as a marker of cardiovascular risk
- CIPN objective measures: Sural nerve amplitude assessed by electrophysiological testing and intraepidermal nerve fiber density measured through skin biopsy

Incorporation of these biomarkers into future clinical studies may enhance patient selection, improve mechanistic understanding, and accelerate the development of effective and targeted chemoprotective strategies.

### 7.3 Regulatory pathway for chemoprotection approval

The U.S. Food and Drug Administration (FDA) has approved several chemoprotective agents, including dexrazoxane, amifostine, and mesna, based on Phase III clinical trials that employed surrogate endpoints rather than long-term clinical outcomes. Examples include cardiac troponin I as a surrogate marker for cardiotoxicity and hematuria as an indicator of hemorrhagic cystitis. For emerging chemoprotective agents, several candidate surrogate endpoints have demonstrated biological plausibility and clinical relevance:

- CIPN: Changes in sural nerve amplitude measured by nerve conduction studies at 3–6 months correlate strongly with clinically apparent neuropathy at 12 months (*r* = 0.71) (Dorsey et al., 2019).
- Cardiotoxicity: The area under the curve (AUC) of cardiac troponin I during anthracycline therapy has shown good predictive performance for subsequent declines in left ventricular ejection fraction (LVEF), with a reported sensitivity of 85% (Cardinale et al., 2004).
- Nephrotoxicity: Early elevations in urinary kidney injury molecule-1 (KIM-1) and neutrophil gelatinase-associated lipocalin (NGAL) within 48 h of treatment have been associated with later increases in serum creatinine and the development of nephrotoxicity (Ichimura et al., 2004).

Although these biomarkers possess strong face validity and are supported by emerging evidence, none has yet undergone formal qualification as a regulatory endpoint by the FDA or the European Medicines Agency (EMA). Ongoing efforts by organizations such as the Critical Path Institute’s Chemotherapy-Induced Toxicity Network (CITN) aim to establish the evidentiary framework required for regulatory acceptance of these surrogate markers, thereby facilitating the development and evaluation of next-generation chemoprotective therapies.

### 7.4 Lessons from negative trials

Not all promising preclinical strategies have translated successfully. Several high-profile failures offer critical lessons for the field:

Calcium/magnesium for oxaliplatin-induced CIPN: Calcium/magnesium infusions showed promise in Phase II (Gamelin et al., 2004) but failed in the Phase III trial by Loprinzi et al. (2014), which randomized 353 patients and found no difference in neuropathy incidence (59% vs. 58%, p = 0.92) (Loprinzi et al., 2014). This failure has been attributed to inadequate Phase II sample size, publication bias favoring positive results, and possible interference with oxaliplatin efficacy. The lesson: Phase II studies with small sample sizes (n < 50) are prone to false positives, and mechanistic plausibility does not guarantee clinical efficacy.
Vitamin E for cisplatin neuropathy: Vitamin E (tocopherols) showed conflicting results in meta-analyses, with some suggesting mild benefit but others finding no clear effect (GBD 2016). The largest randomized trial (n = 108) showed no significant reduction in neuropathy incidence (RR = 0.81, 95% CI 0.58–1.13). The lesson: Heterogeneous study designs and outcome measures prevent reliable meta-analysis, highlighting the need for standardized endpoints.
Acetyl-L-carnitine for CIPN: Acetyl-L-carnitine was associated with increased neuropathy in the placebo arm of one trial, suggesting potential harm. This paradoxical finding highlights that agents with plausible mechanisms can have unexpected effects, underscoring the need for rigorous safety monitoring and adequately powered trials.

Key Lessons Synthesized:

1. Inadequate Phase II sample sizes (n < 50) lead to false positives that fail to replicate
2. Publication bias skews the literature toward positive results, creating an evidence base that overestimates efficacy
3. Mechanistic plausibility does not guarantee clinical efficacy—empirical evidence remains paramount
4. Surrogate endpoints must be validated before Phase III—the calcium/magnesium experience demonstrates that unvalidated surrogates can lead to costly failed trials
5. Safety monitoring must be rigorous—agents with plausible mechanisms may cause harm rather than benefit

### 7.5 Comparative analysis of translational progress

When comparing the translational progress across categories, several patterns emerge. Strategies with simple mechanisms and well-defined endpoints have advanced further: limb hypothermia (mechanical vasoconstriction, clear physiological endpoint) and metformin (repurposed drug with known safety profile) have reached Phase III, whereas nanoformulated natural products with pleiotropic mechanisms remain preclinical. Dual COX-2/TNF-α inhibitors occupy an intermediate position—NO-ASA has early clinical data but no chemoprotection-specific trials, while synthetic inhibitors remain preclinical. Nrf2 modulators show the greatest mechanistic complexity and the most challenging risk-benefit balance, with no clear path to clinical approval for chemoprotection.

This pattern suggests that simplicity in mechanism and endpoint clarity may be more important for clinical translation than theoretical mechanistic elegance. Future development strategies should prioritize clear chemoprotection endpoints, biomarker-driven patient selection, and adequately powered Phase II trials before advancing to Phase III.

## 8 Future directions

### 8.1 Next-generation nanoformulations

Future advances in chemoprotection are likely to be driven by next-generation nanocarriers that enhance drug delivery, tissue selectivity, and patient adherence. Hybrid lipid-polymer nanoparticles offer high drug-loading capacity and sustained release, while active targeting using tumor-associated antigens (e.g., HER2 and EGFR) or microenvironmental cues such as pH and reactive oxygen species (ROS) may enable selective delivery of chemoprotective agents to normal tissues while minimizing tumor exposure. Additionally, oral nanocarrier systems, including chitosan-based nanoparticles and cell-penetrating peptides, have the potential to improve bioavailability, convenience, and long-term adherence to chemoprotective therapies.

### 8.2 Dual-action chemoprotectants

The development of agents capable of simultaneously protecting normal tissues and sensitizing tumor cells to anticancer therapy represents a major goal in chemoprotection research. NO-ASA serves as an important prototype, while other promising candidates include bardoxolone methyl and dimethyl fumarate, both of which activate the Nrf2 pathway and possess anti-inflammatory and antioxidant properties (Hayes et al., 2020). Such agents offer the possibility of improving therapeutic outcomes by reducing treatment-related toxicity without compromising, and potentially enhancing, antitumor efficacy.

### 8.3 Patient selection and precision chemoprotection

Advances in genomics and precision medicine are expected to play an increasingly important role in chemoprotection. Germline and tumor sequencing can help identify patients at elevated risk of specific treatment-related toxicities, such as CYP2C8 variants associated with paclitaxel-induced neuropathy and SLC28A3 variants linked to anthracycline-induced cardiotoxicity (Hertz et al., 2013). Integrating genetic risk profiling with targeted chemoprotective interventions may facilitate personalized prevention strategies and optimize benefit-risk ratios for individual patients.

### 8.4 Emerging concepts

Several emerging concepts have been proposed in the chemoprotection field, including the use of nanovaccines and early-detection platforms integrated with protective interventions. However, these approaches remain largely theoretical and have not yet been evaluated experimentally. Consequently, they are beyond the scope of the present review and warrant future investigation as the field evolves.

### 8.5 Emerging mechanistic frontiers

While the three major strategies discussed above represent the most clinically advanced approaches, several emerging mechanistic areas warrant attention for their potential to open novel chemoprotective avenues.

#### 8.5.1 Ferroptosis inhibition

Ferroptosis, an iron-dependent form of regulated cell death driven by lipid peroxidation, has recently been implicated in chemotherapy-induced organ damage, particularly cardiotoxicity and nephrotoxicity. Doxorubicin has been shown to induce ferroptosis in cardiomyocytes through iron accumulation and lipid peroxidation, while cisplatin causes ferroptotic cell death in renal tubular cells. Preclinical studies have shown that ferroptosis inhibitors, including liproxstatin-1 and ferrostatin-1, can reduce doxorubicin-induced cardiac injury in murine models. Nanoparticle-based delivery of ferroptosis inhibitors represents an emerging Frontier. The intersection with Nrf2 signaling is particularly relevant, as Nrf2 upregulates ferroptosis defense genes (GPX4, FTH1, SLC7A11), suggesting that Nrf2 activators may exert chemoprotective effects in part through ferroptosis inhibition.

#### 8.5.2 Neuroimmune signaling in CIPN

Chemotherapy-induced peripheral neuropathy is increasingly recognized as involving neuroimmune interactions beyond direct neuronal damage. Paclitaxel and oxaliplatin activate spinal cord microglia and astrocytes via CX3CR1 and TLR4 signaling, leading to release of pro-inflammatory cytokines (TNF-α, IL-1β, IL-6) that sensitize nociceptive pathways. Targeting these neuroimmune pathways with minocycline (microglial inhibitor) or pentoxifylline (TNF-α inhibitor) has shown promise in animal models, though clinical translation remains limited. The relevance to the present review is twofold: (i) neuroimmune signaling represents an extension of the inflammatory mechanisms targeted by COX-2/TNF-α inhibitors, and (ii) these pathways may be amenable to modulation by nanoformulated agents designed to cross the blood-nerve barrier.

#### 8.5.3 Pyroptosis and gasdermin-mediated toxicity

Pyroptosis, a pro-inflammatory form of programmed cell death mediated by gasdermin proteins, has recently been linked to chemotherapy-induced tissue injury. Cisplatin has been shown to activate NLRP3 inflammasome and caspase-1 in renal tubular cells, leading to gasdermin D cleavage and pyroptosis. Similarly, doxorubicin-induced cardiotoxicity involves NLRP3 inflammasome activation. Pharmacological inhibition of NLRP3 (e.g., with MCC950) or gasdermin D represents a potential strategy for nephroprotection and cardioprotection. The pathway intersects with dual COX-2/TNF-α inhibition, as inflammasome activation is regulated by inflammatory signaling, suggesting that dual inhibitors may exert effects upstream of pyroptosis.

#### 8.5.4 Integrated perspective on emerging mechanisms

These emerging mechanisms share common features with the established pathways discussed above. Ferroptosis is intimately linked with oxidative stress (Nrf2 pathway), neuroimmune signaling overlaps with inflammatory mediators (COX-2/TNF-α pathway), and pyroptosis involves inflammasome activation that could be targeted by dual inhibitors. Future chemoprotective strategies may need to address multiple pathways simultaneously, possibly through rationally designed combination therapies or multi-target agents. The development of agents that simultaneously modulate Nrf2, inhibit ferroptosis, and suppress neuroimmune signaling represents a long-term goal, though substantial preclinical validation is required.

## 9 Conclusions and integrative summary

This review highlights the growing potential of emerging chemoprotective strategies to reduce treatment-related toxicity while preserving anticancer efficacy. When viewed collectively, the three major categories of emerging chemoprotective agents address complementary but distinct mechanistic dimensions of chemotherapy-induced injury:

- Nanoformulations solve the pharmacokinetic barrier that has historically limited natural products, enabling these pleiotropic antioxidants to reach therapeutic concentrations at sites of toxicity. However, most studies have focused on antitumor efficacy rather than true chemoprotection, representing a fundamental disconnect between preclinical development and clinical need.
- Dual COX-2/TNF-α inhibitors target the inflammatory cascade that amplifies tissue damage following initial cytotoxic insult. NO-ASA demonstrates encouraging preclinical and early clinical activity but requires further evaluation of long-term safety and tolerability, and no chemoprotection-specific trials have been initiated.
- Nrf2 modulators engage the endogenous antioxidant response, enhancing the capacity of normal cells to withstand oxidative injury. Sulforaphane has shown promise in small trials for nephroprotection, but the tumor protection risk remains a significant barrier to clinical translation.
- Repurposed agents and physical interventions including metformin and limb hypothermia represent practical approaches with favorable safety profiles and emerging clinical evidence, with limb hypothermia currently the only strategy in Phase III for chemoprotection. However, definitive conclusions regarding their effectiveness await results from ongoing randomized trials.

Several themes emerge from comparing translational progress across categories:

1. Simple mechanisms and clear endpoints advance faster. Limb hypothermia (mechanical vasoconstriction with measurable physiological endpoint) has reached Phase III, whereas pleiotropic natural products remain preclinical.
2. Failure cases reveal critical lessons. Calcium/magnesium for CIPN and vitamin E for cisplatin neuropathy failed in Phase III despite promising Phase II data, highlighting the need for adequately powered trials with validated endpoints and the recognition that mechanistic plausibility does not guarantee clinical efficacy.
3. The Nrf2 paradox remains unresolved. Nrf2 activators (sulforaphane, curcumin) protect normal tissues but risk protecting tumors, while Nrf2 inhibitors (brusatol) sensitize tumors but increase normal tissue toxicity. Nanoformulation strategies may offer a solution through selective delivery, but this requires preclinical proof-of-concept.
4. Biomarker-driven patient selection is essential. Genetic variants (CYP2C8 for paclitaxel neuropathy, SLC28A3 for anthracycline cardiotoxicity) and pathway-specific markers (NQO1 activity, PGE_2_ levels, sural nerve amplitude) could enable personalized chemoprotection, but these biomarkers require prospective validation.
5. Mechanistic convergence offers opportunities for combination strategies. Nrf2 activation suppresses NF-κB (complementing COX-2/TNF-α inhibition), inhibits ferroptosis (emerging pathway), and reduces inflammation. Dual-action agents or combination nanoformulations could achieve synergistic protection while minimizing individual drug doses and toxicity.

The optimal chemoprotective strategy may ultimately combine elements from all three approaches: a nanoformulated agent that delivers both an anti-inflammatory and an Nrf2-activating payload to normal tissues while avoiding tumor protection. This “dual-mechanism” approach could theoretically achieve synergistic protection while minimizing individual drug doses and toxicity. However, such combination strategies require extensive preclinical optimization and rigorous clinical validation.

Overall, future progress in chemoprotection will depend on biomarker-driven patient selection, validated surrogate endpoints, advanced drug-delivery systems, and well-designed clinical trials. The ultimate goal remains the development of interventions that selectively protect normal tissues without compromising anticancer efficacy, thereby improving treatment outcomes and quality of life for patients undergoing cancer therapy.
