# Supplementary material for: A narrative review of emerging chemoprotective agents for chemotherapy-induced toxicity: nanoformulated natural products, dual COX-2/TNF-α inhibitors, and Nrf2 modulators
Source: Front Pharmacol. 2026 Sep 3;17:1907919. doi: 10.3389/fphar.2026.1907919 (PMC13582253; doi:10.3389/fphar.2026.1907919)
Supplement: Supplementary file 1 [file DataSheet1.docx]

**SUPPLEMENTARY MATERIAL**

**Supplementary Table S1. Evidence Grading of Emerging Chemoprotective Agents**

| **Agent** | **Category** | **Highest Level of Evidence*** | **Key Evidence** | **Major Limitation** | **Justification** |
| --- | --- | --- | --- | --- | --- |
| Curcumin nanoparticles | Nanoformulated natural products | IV | Multiple animal models showing reduced chemotherapy toxicity and improved bioavailability | No completed Phase III trials | Strong preclinical evidence but limited clinical validation |
| Resveratrol nanoparticles | Nanoformulated natural products | IV | Enhanced pharmacokinetics and antioxidant activity in animal studies | Lack of large human studies | Evidence restricted largely to preclinical models |
| EGCG nanoformulations | Nanoformulated natural products | IV | Demonstrated antioxidant and anti-inflammatory effects *in vivo* | Limited clinical translation | Predominantly preclinical evidence |
| Luteolin nanocapsules | Nanoformulated natural products | IV | Improved anticancer efficacy in xenograft models | No human chemoprotection trials | Animal evidence only |
| Naringenin HA nanoparticles | Nanoformulated natural products | IV | Selective tumour targeting and reduced tumour incidence in rodents | No clinical studies | Preclinical proof-of-concept |
| NO-ASA | Dual COX-2/TNF-α inhibition | III | Phase I human trial in FAP plus extensive preclinical studies | Small uncontrolled clinical trial | Early clinical evidence available |
| Synthetic pyrazole dual inhibitors | Dual COX-2/TNF-α inhibition | V | In vitro and anti-inflammatory animal studies | No tumour or chemoprotection studies | Mechanistic evidence only |
| Sulforaphane | Nrf2 activator | IV | Multiple animal studies showing protection against chemotherapy toxicity | Potential tumour-protective effects | Strong biological rationale but limited clinical evidence |
| Curcumin (Nrf2 pathway modulation) | Nrf2 activator | IV | Preclinical protection against oxidative injury | Poor bioavailability in native form | Evidence mainly preclinical |
| Brusatol | Nrf2 inhibitor | IV | Enhanced chemotherapy response in xenograft models | Increased normal tissue toxicity | Preclinical chemosensitization evidence |
| Metformin | Drug repurposing | III | Large retrospective cohorts and ongoing Phase II trial | Evidence largely limited to diabetic populations | Human observational evidence available |
| Limb hypothermia | Physical intervention | II–III | Prospective studies and ongoing Phase III trials | Long-term effectiveness under investigation | Most clinically advanced emerging strategy |

*Evidence Levels: I = Multiple Phase III randomized trials; II = Phase II randomized trials; III = Observational human studies or Phase I trials; IV = Animal studies; V = In vitro/mechanistic evidence.

**Supplementary Table S2. Database Search Strategies**

| PubMed | ("chemoprotection" OR "chemoprotective agent*" OR "treatment-related toxicity") AND ("cancer" OR "oncology" OR "chemotherapy") AND ("nanoparticle*" OR "nanocarrier*" OR "nanoformulation*" OR "curcumin" OR "resveratrol" OR "EGCG" OR "luteolin" OR "naringenin" OR "NO-ASA" OR "nitric oxide donating aspirin" OR "Nrf2" OR "sulforaphane" OR "brusatol" OR "metformin" OR "limb hypothermia")  Scopus  TITLE-ABS-KEY ( ("chemoprotection" OR "chemoprotective agents") AND ("cancer" OR "chemotherapy") AND ("nanotechnology" OR "nanoparticles" OR "natural products" OR "Nrf2" OR "metformin" OR "hypothermia") ) |
| --- | --- |
| Web of Science | TS=( ("chemoprotection" OR "chemoprotective agent") AND ("cancer" OR "chemotherapy") AND ("nanoformulation*" OR "natural product*" OR "NO-ASA" OR "Nrf2 modulator*" OR "metformin") ) |
| ClinicalTrials.gov | Condition/Disease: Cancer  Other Terms: chemoprotection OR chemotherapy toxicity OR peripheral neuropathy OR cardiotoxicity OR nephrotoxicity OR metformin OR hypothermia OR curcumin OR Nrf2  Filters: Completed, Recruiting, Active-not recruiting, Interventional studies, Adult populations  Search period: Database inception to May 2026  Language restriction: English  Document types: Original research articles, clinical trials, systematic reviews, meta-analyses, and relevant preclinical studies. |

**Supplementary Figure S1.** Literature identification and selection process for the narrative review
